# Annoyance in Response to Vibrations from Railways

**DOI:** 10.3390/ijerph15091887

**Published:** 2018-08-31

**Authors:** Laura Maclachlan, Mikael Ögren, Elise van Kempen, Laith Hussain-Alkhateeb, Kerstin Persson Waye

**Affiliations:** 1 Department of Public Health and Community Medicine, University of Gothenburg, Box 414, 40530 Göteborg, Sweden; mikael.ogren@amm.gu.se (M.Ö.); laith.hussain@gu.se (L.H.-A.); kerstin.persson.waye@amm.gu.se (K.P.W.); 2 Centre for Sustainability, Environment and Health (RIVM), P.O. Box 1, 3720BA Bilthoven, The Netherlands; elise.van.kempen@rivm.nl

**Keywords:** annoyance, vibration, rail, train, railway proximity

## Abstract

Rail transport is a key stepping stone in the EU’s transport policy and is pinpointed for investment and growth over the coming decades. This expanding infrastructure implies increased exposure to environmental stressors, such as noise and ground-borne vibrations. Little is known about the health impacts of exposure to these vibrations. The aim of this paper is to examine the association between annoyance from rail vibrations and the distance of residential dwelling from the railway. It reports the first results of a large epidemiological study, EpiVib, which was designed to investigate the long-term health effects of exposure to rail vibrations. The first part of this study examines a self-reported questionnaire. In total, 6894 individuals aged between 18 and 80 living within 1 km of a railway in west Sweden participated. Results presented here examine the association between distance to the railway and annoyance from vibrations and are stratified by train type. A positive association between closer distance and increased annoyance is seen. After adjustment for important modifiers, results showed that vibrations from freight trains and maintenance operations are reported to be moderately and highly annoying at distances of up to 400 m from the railway and diesel up to 300 m. Vibration from passenger and fast trains are significantly annoying up to 200 m from the track. Vibration from freight trains and maintenance operations were considered highly annoying up to 300 m from the track, diesel up to 400 m. Vibration from passenger and fast trains are not reported to be highly annoying after adjustment. Heavier, slower moving locomotives, in the form of diesel and freight trains, appear to be the source of annoyance at distances further from the railway compared to passenger and fast trains. This has implications in terms of property, transport, and infrastructure planning.

## 1. Introduction

Rail transport is heralded as a sustainable approach that can contribute to a reduced climate burden. The European Union has highlighted this form of transport as an area for expansion over the coming decades and it is a core component of the development of the trans-European transport network [1]. There are far-reaching public health benefits of this development; however, populations living in close proximity to railways are impacted by its infrastructure. These impacts can be positive, for example being close to transport networks, but also have negative implications for those living close to the railway, both in the shorter and longer term in terms of noise, vibrations, and air pollution [2,3]. The Swedish Transport Administration recommends a maximum vibration level in residential buildings of a weighted RMS value of 0.4 mm/s [4]. It is estimated that in Sweden approximately 54,000 people are exposed to railway vibrations higher than this and at risk of disturbance [5,6]. This is a picture seen in other countries. An example of this is the Netherlands where approximately 1,350,000 residents over age 16 live within 300 m of a railway and are at risk of vibration exposure [7,8].

Health and well-being effects of noise from rail transport have been investigated since the 1970s. One of the principle effects investigated is annoyance. During the past decades, several exposure-response relationships have been derived for the association between railway noise and (severe) annoyance [9]. The recently published WHO evidence review on annoyance found indications that residents’ annoyance from a given rail traffic noise level increases with time [9].

The WHO evidence review examined cardiovascular and metabolic effects of environmental noise and demonstrated that several published studies investigated the impacts of rail traffic noise. Most of these studies were of a cross-sectional design. The results of these studies did not show a consistent picture: in relation to ischemic heart disease and stroke, positive but statistically non-significant effects were found; in relation to diabetes, unexpectedly negative effects were found with rail traffic noise decreasing the risk of diabetes. For hypertension, the results were inconsistent [10]. In 2014, it was estimated that, in total, about 7.5 million residents in Europe have an exposure of 55 dB (LDEN) or higher to noise from railways. More than 1.1 million adults living in areas at levels equal to or above 55 dB (LDEN) are annoyed by noise from rail traffic noise; 400 thousand are severely annoyed. Railway noise exposure also is estimated to lead to 380 thousand sleep disturbed adults. The total number of hospital discharges due to rail traffic noise-related coronary heart disease and stroke is almost 4000 per year [11].

In the area of noise and health, annoyance is an often-used end point that indicates how people perceive and appraise the noise. Annoyance is a broad term used to describe an individual’s negative assessment of factors in the surrounding environment [12]. It can be defined as “a feeling of displeasure associated with any agent or condition, known or believed by an individual or group to adversely affect them” [13]. Environmental annoyance to factors in an individual’s surroundings can be significant, particularly as exposure can occur in environments in which considerable proportions of time are spent and which may be without individual control to change. Stressors that are intractable, where there is a perception that little can be done to alter or remove the source, can exacerbate the negative feelings [14,15].

Environmental annoyance has been well-documented in relation to noise [16], but the effects of vibrations are less well-studied to date. In the few available studies, an association was found between indicators of exposure to vibration caused by the rail traffic. Freight trains were shown to be most annoying. However, these studies had a relatively small sample size and were not always able to look into the role of situational and demographic factors such as the concern of property damage and expectation of future vibrations. Licitra et al. conducted a larger study that investigated noise and vibration annoyance. Age of the building did affect vibration annoyance, with those in older buildings reporting higher annoyance [17].

Studies show that vibrations from rail transport can be a significant source of annoyance. Peris et al.’s questionnaire study of 931 residents living within 100 m of a railway in England showed that annoyance due to railway vibration was significant and differed depending on the time of day, with greatest annoyance during evenings and at night [18]. The recent European Cargovibes project examined data from a number of countries in Europe and also found that evening and night-time vibration was associated with greater annoyance [19]. Some of the project findings were, however, different across countries. In the Dutch study population (*n* = 156), residents reported annoyance in response to vibrations with a positive dose–response relationship, in particular with regard to freight trains. However, in areas with high vibration levels in Poland, annoyance due to railway vibrations was reported as being relatively low with no clear dose–response relationship (*n* = 104). This implies that situational, personal, and demographic factors may have an important role to play. It was suggested that the perceived importance of freight trains in Poland tempered annoyance and, in contrast, that concern about property damage at sites in the Netherlands augmented annoyance. It is possible that vibration levels were lower in the Polish cohort or that other factors not measured had an important modifying effect. Nevertheless, situational and demographic factors do appear to have an important role to play.

In Norway, Klaeboe and Fyhri studied annoyance in relation to estimated road and railway vibration levels [20]. With exposure to an estimated vibration velocity of approximately 0.5 mm/s, 10% of the respondents reported being highly annoyed by vibrations from road and rail sources and 40% reported being annoyed. There was no significant difference in annoyance depending on the source of vibration, whether road or rail. They found a dose–response relationship between the percentage of individuals reporting annoyance and vibration velocity. Nevertheless, not all individuals reacted to similar vibration levels in the same way, which also suggests that individual, situational, and demographic factors are significant and have an important role to play in changing individual perception of and reaction to vibrations.

Research conducted in the United States as part of the Transit Cooperative Research Program D-12 showed a positive dose–response relationship between vibration/noise exposure and annoyance [21]. Furthermore, an interaction effect has been seen with noise and vibrations, with increased annoyance from exposure to both vibrations and noise [22].

Another problem faced in studies investigating the effects of vibrations due to rail traffic is that it is very difficult to assess the vibration exposure in a reliable way. Studies largely have to make use of rather expensive vibration measurements. Valid models that can be used to assess the exposure to vibrations are currently scarce. Distance to the railway track appears to be the best proxy for exposure to vibrations from rail traffic [8,23].

The aim of this paper is to examine the relationship between distance from the railway and annoyance. In this first analysis of data in the EpiVib study, distance is used as a proxy for vibration exposure levels. Estimating vibration levels in properties is complex and the best algorithms are yet to be identified. Models are currently being developed and one such model is being developed as part of the EpiVib study. Through the Swedish Transport Administration, approximately 2000 ground-borne vibration measures and 700 comfort measures have been accessed. These will be used to develop a model that builds on previously developed models [5,24].

This paper presents the results from the EpiVib cohort. This study was designed to understand the long-term health impacts of exposure to vibrations from rail and to gain more insight into the situational and demographic factors that play a role. The study was conducted in the Västra Götaland, Värmland, and Örebro regions of Sweden in 2017. The aim of this first paper is to ascertain the association between distance from the railway and residents’ self-reported annoyance from vibrations caused by different train types. It is cross-sectional in nature. The intention of the wider study is to follow-up the cohort over time, which is a considerable strength. The main hypothesis is that closer proximity to the railway will be associated with a higher degree of annoyance as exposure to vibrations will be greatest. A further hypothesis is that the degree of annoyance will vary by train type and that freight trains will be considered the most annoying as previous noise research reports that freight trains are more annoying than other train types [25]. A similar pattern in relation to vibrations is anticipated.

## 2. Materials and Methods

### 2.1. Study Design and Sample

The study population was randomly selected from residents living close to the railway in the Västra Götaland, Värmland, and Örebro regions of Sweden. The areas were identified using geographic information systems (GIS) and were within 1 km of a railway in use. Selected areas (i) had a minimum of ten passing freight trains per day and night, (ii) were areas in which vibrations measurements had been taken in a number of dwellings, and (iii) were populated and with no major motorways or air traffic nearby. Addresses were obtained from the Swedish land registry and individuals residents at these addresses were identified via the Swedish National Board of Health and Welfare (NAVET). Geographic areas were selected to include a population exposed to vibrations from rail and a control population with a range of vibration and noise level exposures.

Ethical permission to investigate the relationship between annoyance and health via a self-reported questionnaire and exposure to vibrations from rail traffic was sought with the Swedish Central Ethical Review Board (Etikprövningsnämnderna) in Gothenburg. Ethics permission was granted in August 2016 (DNR 662-16). The study was conducted in accordance with the rules of the Declaration of Helsinki of 1975 [26]. All participants signed informed consent forms.

Residents between the ages of 18–80 years were included. Up to two people in each household were randomly selected using a computer program. In total, 35,011 individuals were asked to participate in the study. Two reminders were sent. Data were collected between March and June 2017.

### 2.2. Development of the Questionnaire

Self-reported questionnaires were completed. In order to increase the response rate, participants had the opportunity to fill in a written questionnaire or an online questionnaire. Questions were informed from previous research [3,8,18] and a qualitative study conducted as part of the EpiVib project [27], and were piloted in October 2016. Three hundred questionnaires were sent to residents within 50 m of a railway in Borås municipality and a 21% response rate was achieved. A Cronbach alpha assessment was performed to assess internal consistency of the questionnaire items.

The questionnaire contained several blocks of questions. The first block focused on perception and annoyance in relation to vibrations specifically from rail sources. Other traffic sources were also touched upon. In the second block, questions were then asked in relation to noise, as an important modifier. A range of health outcomes were included with a focus on sleep disturbance, medical diagnoses, somatic symptoms, as well as coping mechanisms. Information regarding the property, such as type, construction, and triple glazing, were included. Finally, sociodemographic information, such as education level, income, physical activity, waist circumference, and lifestyle, was obtained.

There is variation in weight, speed, and length of the different train types that run in the study areas making it difficult to provide a complete view of the specific technical details. In general, the majority of passenger and fast trains are electric with a small proportion that have diesel engines. Fast trains are passenger trains with a maximum speed of 200 km/h. Freight trains have an approximately equal distribution of diesel and electric engines. The maximum allowed length for freight trains is approximately 700 m, and the average length is around 400 m. Maintenance operations are predominantly powered by diesel locomotives but may also include freight trains. 

Distance from railways was used as a continuous variable to examine annoyance scores. Distances were also categorized into seven 100-m intervals with an eighth category designed into a 300m interval (≤100, 100–200, 200–300, 300–400, 400–500, 500–600, 600–700, 700–1000 m). The final category is wider than the others and is used as a control group, as the effects of vibrations are not expected to be perceived at this distance.

Questions regarding the building were included, such as type of house (detached, terraced, etc.), type of foundation (cellar, directly onto the ground), floor construction (concrete, wood, etc.) and whether insulation measures, such as triple glazing, had been made.

Participants were asked how often they noticed noise from rail traffic. Answers were as follows: never/seldom; at least once a year; at least once a month; at least once a week; two to three times a week; every day.

Annoyance was measured as part of a self-administered questionnaire by means of standard questions. The following wording was used: “Over the last 12 months how annoyed have you been by vibrations from different trains”. Answers were indicated on an 11-point Likert scale and were given independently for each different train type. Participants were asked to rate their annoyance from train vibrations on an 11-point scale from 0 (not at all annoyed) to 10 (highly annoyed). This scale is standardized for noise annoyance in ISO/TS-15666 [28]. There was also an option to answer that vibrations were not noticed. If participants indicated not noticing the vibrations, annoyance was recoded to 0 (not at all annoyed). The results of the annoyance questions were subsequently dichotomized, with 5–10 defined as being annoyed and 8–10 as being highly annoyed (HA).

Sex and age of participants was obtained. Questions were asked about other potentially important factors identified in previous studies [29], including worry and an expectation of changes in future vibration levels. Socioeconomic information was obtained, including sex, age, and household income.

Four questions about worries were asked. They concerned worry over the preceding twelve months regarding the effects of vibrations on their own health, damage to property and belongings, safety about living close to the railway, and negative effect on property value. They were answered on an 11-point Likert scale, with 0 as *not at all worried* to 10 *extremely worried*.

Participants were asked about their expectation of future vibrations and could answer that they would *increase*, *decrease*, *remain the same*, or *do not know*.

### 2.3. Statistical Analysis

Response rate and discussion regarding non-respondents can be found in the Supplementary Materials.

The associations between distance from railways and inhabitants’ self-reported (high) annoyance were examined independently for each train type (passenger, freight, fast, diesel, and maintenance). The exposure group was stratified by 100 m distances within 700 m. The control group, which is comprised of participants between 700 and 1000 m from the railway, was included as a one-strata forming the reference group in the odd ratio (OR) analysis. It is suggested in general that vibrations are not noticeable at distances over 300 m from the railway [23]. In this analysis this hypothesis was tested by examining distances beyond 300 m. Logistic regression was used to assess these associations, both at crude and multivariate levels, utilizing a list of qualified co-variates from the questionnaire. A likelihood ratio test was used to determine best-fit multivariate models. Respondent age and sex information were retained in the multivariate models, although they did not have a significant contribution to the model. STATA/SE 14.2 (StataCorp. L.C.C., College Station, TX, USA) was used for the analysis. Crude and adjusted results are included. Factors that were likely to significantly influence the association between annoyance and distance from the railway or contribute to explaining the statistical model were adjusted for using multivariate logistic regression, as indicated in models 1–10. Noise perception had the largest confounding effect on annoyance and was therefore adjusted for first in model 1. The following factors were adjusted for: noise perception, sex, age, worry about health, worry about safety, worry about home, vibration expectation, household income, and years resident at the property.

## 3. Results

The overall response rate of individuals completing a questionnaire was 21.9% (7679 individuals). Of these, 6894 (19.7%) completed the questionnaire, gave permission, and had complete data for the variables and are included in the analysis. The response rate differed by distance from the railway and is discussed in Supplementary Materials.

There was an even distribution of men and women in the respondents, as seen in Table 1. The age range was 18–80 years, with an average of 55.8 years. This study population comprised a higher proportion of individuals with further education compared to the general population. Respondents were in general well educated with 41.4% having completed a university degree compared to 21.8% that had completed elementary school. This compares to 25.8% of the adult population in Västra Götaland that have more than three years of additional study after school and 12% with elementary school education [30].

In total, 76.1% had a household income of over 30,000 Swedish kronor per month (approximately 3000 euros).

The predominant expectation in terms of exposure to vibrations was that it will remain the same in the future (54.7% of respondents). Very few thought that vibration exposure from rail will decrease (2.4%), and approximately one fifth 17.5% thought that exposure will increase. In general, worry about the effects of vibration exposure was low. Of those that did worry, the main concern was that the property value would be negatively affected, where 6.3% of participants felt this was a problem. This was followed by worry about property damage (3.5%). General safety in terms of living close to the railway and worry about the health effects of vibration exposure were reported by only a small percentage of participants (2.2% and 1.4% respectively).

Almost all (89.6%) respondents owned their own home and most of these properties were detached (see Table 2 for property characteristics). The majority of houses had floors that were constructed of wood (60.9% of living rooms and 67.2% of bedrooms). Respondents had lived at the property for an average of 20.2 years and most (84.7%) had lived there for over five years.

### 3.1. Prevalence of Annoyance and High Annoyance from Vibrations

**Figure 1:**
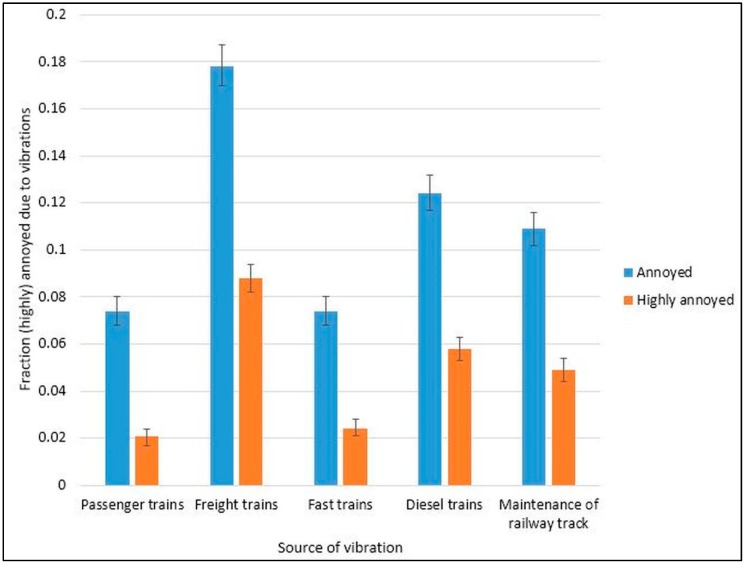
The prevalence of annoyance and high annoyance due to vibrations from the railway track.

The fraction of annoyance and HA by train type is shown in Figure 1. Freight trains were reported as annoying and HA by the most number of participants, and passenger and fast trains by the least.

### 3.2. Annoyance from Vibrations

**Figure 2:**
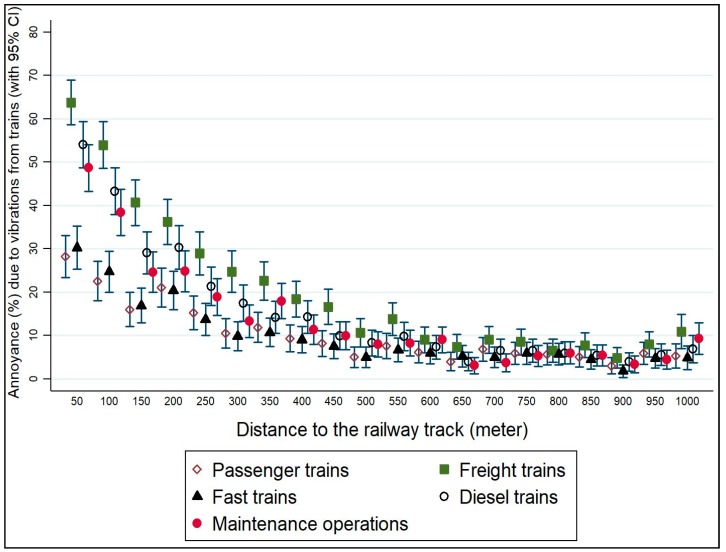
The association between distance from the railway track and annoyance due to vibrations from trains.

The annoyance score by train type and distance from the railway is shown in Figure 2. The most annoying vibration sources were freight trains, followed by diesel trains and track maintenance. The least annoying and HA trains were fast and passenger.

Figure 2 shows the relation between distance from the railway track and annoyance from vibrations for different train types. The figure shows distance to the railway being correlated with the percentage of participants reporting annoyance due to vibrations from trains. Freight trains are reported as the most annoying train type at all distances, and passenger and fast trains the least annoying. There is a positive correlation between proximity to the railway and annoyance from vibrations with those closest reporting being the most annoyed. There appears to be a trend at 300 m from the railway where respondents begin to get more annoyed by vibrations, characterized by a marked increase in the percentage of respondents being annoyed. Freight trains show this trend at a distance of 500 m. 

The OR of being annoyed by vibrations is reported in Table 3 and stratified by distance up to 500 m. Distances over 500 m were not statistically significant and are therefore not reported. Results shown are crude and adjusted for all modifiers. A full table showing all models is found in the Supplementary Materials Table S2. Data for passenger, freight, fast, and diesel trains, and maintenance operations is given. 

The odds of being annoyed by train vibrations increased significantly with closer proximity to the railway (Table 3). Crude results show that all trains types were statistically significantly annoying compared to the control group of those living >700 m from the railway. The association between distance and annoyance was strongest for freight trains, followed by diesel and maintenance. Passenger trains had the weakest association.

After adjustment for all factors, all train types were reported as being statistically significantly annoying; however, the distance with a significant association varied. Within 100 m, maintenance, diesel, and freight were associated with greatest annoyance, with increased odds of 5.8 (95% CI 4.0–8.5), 5.3 (3.6–7.6) and 5.3 (3.8–7.4) respectively. Passenger and fast trains were reported as the least annoying (1.7 (1.2–2.6) and 2.3 (1.5–3.6), respectively). There was a positive correlation between proximity to the railway and increasing annoyance for all train types. Freight trains and maintenance were significantly annoying at distances of up to 400 m. Diesel trains were significantly annoying at distances of up to 300 m. Passenger and fast trains were significantly annoying at distances of up to 200 m.

The strength of association between the perception of rail noise and annoyance was high for all train types, as indicated by the reduction in OR between model 1 and model 2 (see Supplementary Materials). After adjustment for noise perception, the distance at which annoyance was reported significantly reduced for all train types, although at different distances. For example, freight trains were significantly annoying at distances of up to 400 m before adjustment and up to 300 m after adjustment for noise perception.

### 3.3. Role of Demographic and Situational Factors

When included in the statistical model, sex and age did not have a significant effect on the odds of being annoyed (models 3 and 4). There was an association between worry and annoyance from railway vibrations (models 5, 6, and 7). This was true for the three questions asked: worry about health, property, and general safety of living close to the railway line. An expectation of increased vibration exposure (model 8) was significantly associated with increased annoyance. Higher household income was associated with lower annoyance. A longer duration of time the resident had lived at the property had no significant effect on annoyance from passenger train vibrations but was strongly associated with annoyance from freight trains. A longer residential time at the property was associated with slightly higher vibration annoyance.

### 3.4. Highly Annoyed from Vibrations

**Figure 3:**
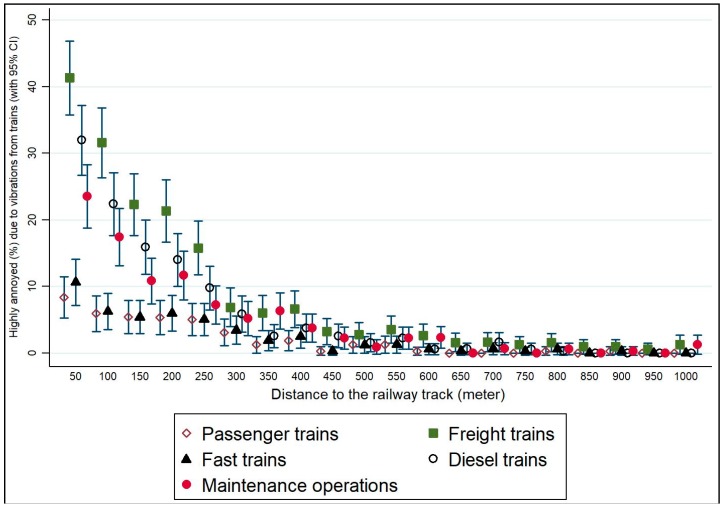
The association between distance from the railway track and high annoyance due to vibrations from trains.

Highly annoyed is more often used as an indicator for policy. Distance in relation to percentage of respondents who reported being HA by train type has therefore been examined (Figure 3).

Freight and diesel trains and maintenance operations are reported as more HA than passenger and fast trains. This is statistically significant at closer distances.

The OR of being HA by vibrations is reported in Table 4 by distance. Data for passenger, freight, fast, and diesel trains, and maintenance is given. Results shown are crude and adjusted for all modifiers. A full table showing all models is found in the Supplementary Materials Table S3. Data for passenger, freight, fast, and diesel trains, and maintenance is given.

The odds of being HA by train vibrations increased significantly with closer proximity to the railway. Crude results (model 1) show that all trains types were statistically significantly HA compared to the control group of those living >700 m from the railway. Diesel trains were reported as the most HA, followed by maintenance and freight, and all three types were significantly annoying at all distances up to 500 m. The association between distance and HA was strongest for freight trains, followed by diesel trains and maintenance. Passenger trains had the weakest association.

After adjustment for possible confounders, the distance to railway was not statistically significantly associated with HA due to vibrations from passenger and fast trains.

Noise perception increased the odds of being annoyed by rail vibrations. Sex and age did not have a significant effect on the odds of being HA. There was an association between worry and HA from railway vibrations with the exception of the association between worry about health and annoyance from freight train vibrations. An expectation of increased vibration exposure was significantly associated with increased HA. Higher household income was associated with lower HA. A longer time of residence at the property was associated with decreased odds of being annoyed by vibrations from all train types.

## 4. Discussion

Both annoyance and HA were studied in this article. Although the use of HA may be more policy and disease relevant, there are arguments for using annoyance in particular as it enables the inclusion of more cases, thus strengthening the results statistically. The main finding was the association between vibration annoyance and distance up to 400 m for freight trains and maintenance operations after correcting for modifiers. This distance was greater than previously assumed, even for uncorrected data. One explanation for this is the type of ground present in Sweden, which may be softer, such as sand and clay, enabling propagation of low frequency vibration for longer distances.

Annoyance and HA have been associated with poor mental health and both are therefore included here as important health determinants [31].

Meta-analysis conducted by a Dutch team has shown a clear association between vibration exposure and annoyance [23]. This is also seen in the results of the European Union funded Cargovibes project [32].

The National Institute for Public Health and Environment in the Netherlands (RIVM) published the results of a study examining the health effects of exposure to vibration in 2015 [8]. The study population was taken from residents aged from 16 years living within 300m of a railway in the Netherlands. Demographics were comparable to EpiVib’s population. Results show that HA increased with proximity to the railway. The EpiVib study also shows that the odds of being annoyed by rail vibrations increased with closer proximity to the railway.

On the comparison of passenger and freight trains, freight trains were consistently considered more annoying. This is similar to findings of other European studies, where freight trains are associated with higher annoyance scores than passenger trains. Diesel trains and maintenance were also reported as significantly annoying at distances further from the railway than passenger and fast trains. Vibrations from diesel and freight trains, and maintenance were also statistically significantly HA after adjustment, which differed in comparison to passenger and fast trains, which were not. The types of trains that were reported as HA may reflect their characteristics of being heavier and longer. This may enhance vibration amplitude. Increased vibration amplitude has been associated with poorer self-reported sleep quality with increased awakenings and subsequent tiredness, as well as with objective physiological changes such as increased heart rate [33]. Freight trains may also be more likely to run at night and may impact an individual’s ability to sleep, which may mean that individuals are more likely to perceive these trains at night when other environmental noise is minimal, implying that the effects of vibrations are therefore felt at distances further from their source.

Findings of an interview study conducted as part of the EpiVib project may also help to explain why freight trains were considered more HA than other trains types. Participants in this study, residents living within 50 m of the railway, reported that heavier, slower-moving trains were considered more disruptive than faster trains such as passenger or more modern “fast” locomotives [27]. It may be that if a stressor is short-lived, it is viewed less negatively as exposure time is short. Previous studies also suggest that other demographic and situational factors have a modifying effect on reported annoyance scores. This may be exacerbated under otherwise quiet nighttime conditions in rural Sweden.

The Dutch study found that education level, sex, age, worry about property damage, and expectations about future exposure were found to have a significant effect on annoyance. A small number of studies have examined factors that may affect annoyance from vibrations. Wong-McSweeney et al. found that concern of property damage and sensitivity to vibrations from construction of a transport system were two important modifying factors, as were age, ownership, and visibility of the construction work [29,34]. Visibility of the source of the stimulus has also been reported to reduce annoyance in Sweden [27].

Demographics, such as sex and age, did not significantly affect annoyance in the EpiVib study. Worry and expectations were found to be significant. An expectation of a decrease in vibration exposure was also significantly associated, as was hearing train noise often. This may be an indication that measures aimed at mitigating and reducing individual worries and managing expectation may contribute to a reduction in annoyance levels. The presence of two stimuli and stress sources implied greater annoyance. EpiVib explored different aspects of worry, which were significant in the project’s initial qualitative study [27]. Worry about health, property damage, and safety significantly increased the odds of being annoyed and HA by vibrations.

Worry was significantly associated with annoyance score and included all aspects that were in the questionnaire about own health, damage to the home, general safety of living along the railway, and property value. Although the direction of association is difficult to elucidate, as worry and annoyance are interlinked, worry is an important modifying factor. Previous studies have shown that concern for property damage has been an important moderator of annoyance [34], despite vibration exposure below levels shown to result in actual physical damage to the property [35]. This raises the possibility that it is not only the vibrations themselves that are annoying but it may also be that the resultant worry is an indirect source of annoyance.

The World Health Organization (WHO) define health as “a state of complete physical, mental and social well-being and not merely the absence of disease or infirmity” [36]. Annoyance, previously here defined as “a feeling of displeasure associated with any agent or condition, known or believed by an individual or group to adversely affect them” [13], by implication compromises this state of complete well-being. There may, therefore, be long-term negative health implications of exposure to rail vibrations.

Noise perception was used as a proxy for noise exposure. It is likely to be an important modifier as populations exposed to rail vibrations are generally also always exposed to rail noise. It is very difficult to find a population exposed to vibrations alone without noise. Populations living above a rail tunnel may be one such small group, but the nature of the vibrations differ and are therefore not comparable. Therefore, it was important to adjust for this in our statistical models. Noise exposure data was not available for this paper and therefore self-reported perception was used as a proxy.

Train type was an important modifier for annoyance. Providing technical details of each train type is technically challenging as there is variation in speed, weight, and length even, for example, across freight trains. This may mean that comparison across countries is more challenging.

The floor on which residents live may be an important modifier of the way in which vibrations are felt and perceived. In this study population 2.2% of participants lived in apartments and the remaining lived in houses (Table 2). The houses in Sweden typically have one or two floors, and the bedroom for two floor-level houses are usually on the second floor. While correction for floor level hence may be relevant for sleep disturbance, we judged it less obvious for noise annoyance.

### 4.1. Strengths and Limitations

One of the strengths of this Swedish study is its large population. The intention is to follow up this cohort over time, which will enable analysis of the incidence rate. The next stage of the project will be to estimate vibration exposure based on extrapolation of measurements among properties with similar geological conditions. This will enable a better understanding of annoyance in response to vibration exposure. Proximity to the railway alone does not necessarily imply vibration exposure and other factors will be important, such as ground type and noise level. Data from the large EpiVib cohort gives an opportunity to study this on a large scale.

A limitation of the data presented here is that a self-reported questionnaire was used and there will be a degree of reporting and recording bias. A qualitative interview study was conducted prior to the questionnaire being sent. These results are now published [27]. In general, the attitude towards the railway was positive, which may influence the way in which annoyance is reported. This is a cross-sectional study. It is therefore possible that annoyance in general, in other aspects of life, may impact the perception of vibrations and reporting of annoyance in relation to them.

The response rate was low, although higher in populations living closer to the railway. This implies a response bias, as the chance of selective non-response is high and therefore the association between distance and annoyance was overestimated. Basic analysis of non-respondents was conducted. Respondents were statistically significantly older than non-respondents (R 56.2, NR 48.2, *p* = 0.0001) and lived closer to the railway (R 409, NR 460, *p* = 0.0001). This may introduce bias. The effect size was, however, small and unlikely to be significant.

A further limitation is that it was not possible to assess people’s exposure to levels of vibration or noise. This will be possible in the near future.

### 4.2. Implications

Participants reported annoyance and HA from vibrations from different rail sources. This annoyance may have deleterious health effects. It is difficult to evidence the impact that annoyance may have in disease pathogenesis. The second part of the EpiVib study will examine objective health outcomes from register data with the aim to better understand this.

These results may help with infrastructure and transport planning, whereby better knowledge of the distances at which annoying from vibrations occurs, as well as the type of train, can inform building regulations close to the railway and train timetabling.

## 5. Conclusions

This paper aimed to investigate the relationship between distance from the railway and residents annoyance from vibrations from rail sources. There was a positive association between closer distance to the railway and proportion/prevalence of annoyance as well as high annoyance. Train types were an important modifier. Freight and diesel trains, and maintenance were the most annoying and HA and at longer distances. There was an association between vibration annoyance and distance up to 400 m for freight trains and maintenance operations after correcting for modifiers, such as noise annoyance, worry, and vibration expectation. This was further away than previously suggested. Passenger and fast trains were not significantly HA. Lighter, faster trains appear to be associated with lower annoyance levels. Worry about health, safety, and the property, and expectation of increased vibrations were important modifiers of vibration annoyance and may be factors that can be addressed to reduce residents’ annoyance. Better understanding these factors, as well as the distances at which individuals are affected by vibrations by train source, will help to inform transport policy. Transportation authorities may wish to focus on these types of trains as a key step in reducing annoyance and improving residents’ quality of life.

## Figures and Tables

**Table 1:** Descriptive statistics of the participants who completed a questionnaire (*n* = 7679).

Characteristic	%	Average (SD)	*n*
Age		55.8 (15.6)	6894
Women	50.1		6845
*Indicators of socio-economic status*			
Level of education			6834
None	0.8		
Elementary school	21.8		
Gymnasium	36.0		
University	41.4		
Household income ≥30,000 (SEK/month) (approx. 3000 euros)	76.1		6581
Homeowner	89.6		6840
Number of years living at the property		20.2 (14.6)	6795
≥five years	84.7		6787
Expectation of vibration exposure			6847
Will decrease	2.4		
Will increase	17.5		
Will remain the same	54.7		
Worry			
About damage to the property	3.5		6718
That the property value is adversely affected	6.3		6734
About their safety living along the railway track	2.2		6685
About their own health	1.4		6795
Notices noise from trains at least once a week	45.3		6581

Note: In 2016, the median income in Sweden was 29,350 SEK/month.

**Table 2:** Descriptive statistics of the houses of participants who completed a questionnaire.

Building Characteristics	%	N
*Type of building*		7589
Detached	86.6	
Semi-detached	2.2	
Terraced	9.0	
Apartment	2.2	
*Self-reported floor construction*		
Living room		6612
concrete floor only	32.0	
wooden floor only	60.9	
concrete and wooden floor	2.7	
Bedroom		6500
concrete floor only	25.9	
wooden floor only	67.2	
concrete and wooden floor	1.9	
% buildings with triple glazing	49.5	7021
*Property foundations*		
crawlspace (*n*)		1228
cellar (*n*)		2810
concrete slab (*n*)		1523

**Table 3:** Odds of being annoyed by vibrations stratified by distance and train type with moderating factors.

Type of Train	Distance	Crude	After Adjustment for Confounders **
		OR (95%CI)	OR (95%CI)
Passenger	<100	6.2 (4.5–8.4) *	1.7 (1.2–2.6) *
	100–200	3.9 (2.8–5.2) *	1.6 (1.1–2.4) *
	200–300	2.1 (1.5–2.8) *	1.3 (1.0–2.0)
	300–400	1.3 (0.9–1.8)	1.0 (0.7–1.6)
	400–500	1.2 (0.8–1.8)	1.0 (0.6–1.6)
	>700	1	
Freight	<100	18.2 (13.9–23.7) *	5.3 (3.8–7.4) *
	100–200	7.0 (5.4–9.0) *	3.1 (2.2–4–2) *
	200–300	3.0 (2.6–4.3) *	2.0 (1.5–2.8) *
	300–400	1.9 (1.5–2.6) *	1.5 (1.1–2.1) *
	400–500	1.4 (1.0–2.0)	1.1 (0.8–1.7)
	>700	1	
Fast	<100	8.2 (6.0–11.4) *	2.3 (1.5–3.6) *
	100–200	4.5 (3.3–6.2) *	1.9 (1.2–2.8) *
	200–300	2.2 (1.6–3.2) *	1.4 (0.9–2.2)
	300–400	1.6 (1.1–2.3) *	1.3 (0.8–2.0)
	400–500	1.3 (0.9–2.0)	1.1 (0.7–1.9)
	>700	1	
Diesel	<100	16.3 (12.2–21.9) *	5.3 (3.6–7.6) *
	100–200	6.3 (4.7–8.4) *	2.9 (2.0–4.1) *
	200–300	2.9 (2.1–3.9) *	1.8 (1.2–2.6) *
	300–400	1.9 (1.4–2.6) *	1.5 (1.0–2.2)
	400–500	1.4 (0.9–2.0)	1.1 (0.7–1.7)
	>700	1	
Maintenance	<100	15.2 (11.1–20.7) *	5.8 (4.0–8.5) *
	100–200	5.9 (4.3–8.0) *	3.2 (2.2–4.6) *
	200–300	3.1 (2.3–4.3) *	2.1 (1.5–3.1) *
	300–400	2.1 (1.5–2.9) *	1.6 (1.1–2.5) *
	400–500	1.6 (1.1–2.4)	1.4 (1.0–2.3)
	>700	1	

* statistically significant; ** adjusting for noise perception, sex, age, worry about health, worry about safety, worry about home, vibration expectation, household income, and years residing at the property.

**Table 4:** The association between distance and high annoyance due vibrations from different rail traffic sources.

Type of Train	Distance	Crude	After Adjustment for Confounders **
		OR (95%CI)	OR (95%CI)
Passenger	<100	43.6 (10.5–180.1) *	2.4 (0.6–10.7)
	100–200	33.0 (8.0–135.7) *	4.2 (1.0–17.9)
	200–300	10.6 (2.5–45.4) *	2.3 (0.5–10.5)
	300–400	5.0 (1.1–23.5) *	1.3 (0.2–6.8)
	400–500	4.0 (0.8–20.8)	1.6 (0.3–9.2)
	>700	1	
Freight	<100	48.7 (27.6–86.1) *	7.7 (3.8–15.8) *
	100–200	30.0 (12.4–38.7) *	5.9 (2.9–12.1) *
	200–300	5.5 (3.0–10.1) *	2.2 (1.0–4.7) *
	300–400	2.6 (1.3–5.1) *	1.4 (0.6–3.2)
	400–500	2.8 (1.4–5.5) *	2.0 (0.9–4.7)
	>700	1	
Fast	<100	36.1 (11.3–115.9) *	2.1 (0.6–7.3)
	100–200	24.5 (7.7–78.4) *	3.1 (0.9–10.3)
	200–300	8.2 (2.5–27.6) *	2.0 (0.6–7.1)
	300–400	4.2 (1.2–15.3) *	1.1 (0.3–4.6)
	400–500	3.3 (0.8–13.0)	1.4 (0.3–6.2)
	>700	1	
Diesel	<100	212.3 (52.5–859.1) *	22.7 (5.5–94.2) *
	100–200	84.7 (20.9–343.1) *	15.0 (3.6–62.1) *
	200–300	21.9 (5.3–90.8) *	7.1 (1.7–30.1) *
	300–400	10.8 (2.5–46.8) *	4.6 (1.0–20.8) *
	400–500	9.0 (2.0–40.7) *	4.0 (0.8–19.0)
	>700	1	
Maintenance	<100	57.2 (23.3–140.7) *	8.0 (3.1–20.3) *
	100–200	25.6 (10.4–63.2) *	5.8 (2.3–14.7) *
	200–300	11.2 (4.4–28.1) *	4.4 (1.7–11.3) *
	300–400	5.3 (2.0–14.2) *	2.5 (0.9–6.9)
	400–500	3.9 (1.4–11.3) *	2.0 (0.7–6.1)
	>700	1	

* statistically significant; ** adjusting for noise perception, sex, age, worry about health, worry about safety, worry about home, vibration expectation, household income, and years residing at the property.

## Supplementary Materials

The following are available online at http://www.mdpi.com/1660-4601/15/9/1887/s1. Table S1. Overview of response in relation to distance from the railway, Table S2. Odds of being annoyed by vibrations stratified by distance and train type with moderating factors (full version of Table 3), Table S3. Odds of being highly annoyed by vibrations stratified by distance and train type with moderating factors (full version of Table 4).
