# Supplementary material for: Annoyance in Response to Vibrations from Railways
Source: Int J Environ Res Public Health. 2018 Aug 31;15(9):1887. doi: 10.3390/ijerph15091887 (PMC6163999; doi:10.3390/ijerph15091887)
Supplement: Supplementary file 1 [file ijerph-15-01887-s001.pdf]

International Journal of
Environmental Research and Public Health

MDPI

Supplementary Materials: Annoyance in Response to Vibrations from Railways

Pilot study

A pilot study was conducted in October 2016. A total of 300 questionnaires were distributed to 300 households in the municipalities of Bollebygd, Olsfors and Hultafors west of Gothenburg, Sweden. Properties within 50m of the railway were selected. Participants were aged over 18 years. One participant per household was asked to respond. If more than one person was resident in the household, the person with a birthday closest to 18/10/16 (date of printing) to respond.

Sixty-two questionnaires were returned, giving a response rate of 21%.

Response rate main study

Table S1 shows the total response rate and response rate stratified by distance from the railway. In total 7,679 (21.9%) individuals completed a questionnaire. Of these, 6,894 19.7%) gave their written consent to be in the study and are included in the analysis. There was a positive correlation between proximity to the railway and response rate. The highest rate was within 50m of the railway (29.4%). There was a steady decline in response to 16.4% in those individuals living over 500m from a railway.

Table S1. Overview of response in relation to distance from the railway**.**

Distance to the Railway Track (m)	Number of People Invited	Number of People that Returned a Completed Questionnaire	Number of People that Completed the Consent Form
< 50	694	216 (31.1%)	197 (28.4%)
50 – 100	1729	528 (30.5%)	471 (27.2%)
100 – 200	4646	1157 (24.9%)	1027 (22.1%)
200 – 300	4985	1194 (24.0%)	1082 (21.7%)
300 – 500	8089	1870 (23.1%)	1686 (20.8%)
≥500	13,308	2434 (18.3%)	2185 (16.4%)
Unknown	1560	280 (17.9%)	246 (15.8%)
**Total**	**35,011**	**7679 (21.9%)**	**6894 (19.7%)**

Comparison between responders and non-responders showed some selection bias.

Results main study

Table S2. Odds of being annoyed by vibrations stratified by distance and train type with moderating factors (full version of Table 3).

Type of train	Distance	Model 1 OR (95%CI)	Model 2 OR (95%CI)	Model 3 OR (95%CI)	Model 4 OR (95%CI)	Model 5 OR (95%CI)	Model 6 OR (95%CI)	Model 7 OR (95%CI)	Model 8 OR (95%CI)	Model 9 OR (95%CI)	Model 10 OR (95%CI)
Passenger	<100	6.2 (4.5-8.4)*	2.8 (1.9-3.9)*	2.8 (2.0-3.9)*	2.8 (2.0-3.9)*	2.7 (1.9-3.8)*	2.4 (1.7-3.5)*	2.1 (1.4-3.1)*	2.0 (1.3-2.9)*	1.7 (1.2-2.6)*	1.7 (1.2-2.6)*
	100-200	3.9 (2.8-5.2)*	2.2 (1.6-3.1)*	2.2 (1.6-3.1)*	2.2 (1.6-3.0)*	2.0 (1.4-2.9)*	2.0 (1.4-2.8)*	1.7 (1.2-2.5)*	1.7 (1.2-2.5)*	1.6 (1.1-2.3)*	1.6 (1.1-2.4)*
	200-300	2.1 (1.5-2.8)*	1.4 (1.0-2.0)*	1.4 (1.0-2.0)	1.4 (1.0-2.0)	1.4 (1.0-2.0)	1.4 (1.0-2.1)	1.4 (1.0-2.1)	1.4 (1.0-2.1)	1.3 (1.0-2.0)	1.3 (1.0-2.0)
	300-400	1.3 (0.9-1.8)	0.9 (0.7-1.4)	1.0 (0.7-1.4)	1.0 (0.7-1.4)	1.0 (0.7-1.5)	1.0 (0.7-1.6)	1.1 (0.7-1.6)	1.1 (0.7-1.6)	1.0 (0.7-1.6)	1.0 (0.7-1.6)
	400-500	1.2 (0.8-1.8)	0.9 (0.6-1.5)	1.0 (0.6-1.4)	1.0 (0.6-1.5)	1.0 (0.6-1.5)	1.0 (0.6-1.5)	1.0 (0.6-1.5)	1.0 (0.6-1.6)	1.0 (0.6-1.6)	1.0 (0.6-1.6)
Freight	<100	18.2 (13.9-23.7)*	6.9 (5.1-9.2)*	6.9 (5.1-9.2)*	6.9 (5.1-9.2)*	6.8 (5.0-9.2)*	6.5 (4.8-8-9)*	6.0 (4.4-8.2)*	5.6 (4.1-7.7)*	5.2 (3.7-7.3)*	5.3 (3.8-7.4)*
	100-200	7.0 (5.4-9.0)*	3.6 (2.6-4.7)*	3.6 (2.7-4.7)*	3.5 (2.7-4.7)*	3.5 (2.6-4.6)*	3.4 (2.5-4.5)*	3.0 (2.3-4.1)*	3.0 (2.2-4.0)*	3.0 (2.2-4.1)*	3.1 (2.2-4-2)*
	200-300	3.0 (2.6-4.3)*	2.1 (1.6-2.8)*	2.1 (1.6-2.8)*	2.1 (1.6-2.8)*	2.1 (1.6-2.9)*	2.2 (1.6-2.9)*	2.1 (1.5-2.8)*	2.1 (1.5-2.8)*	2.0 (1.5-2.8)*	2.0 (1.5-2.8)*
	300-400	1.9 (1.5-2.6)*	1.3 (1.0-1.8)	1.3 (1.0-1.8)	1.3 (1.0-1.8)	1.4 (1.0-1.9)	1.4 (1.0-2.0)	1.4 (1.0-2.0)	1.5 (1.1-2.1)*	1.5 (1.0-2.1)*	1.5 (1.1-2.1)*
	400-500	1.4 (1.0-2.0)	1.1 (0.8-1.5)	1.1 (0.8-1.5)	1.1 (0.8-1.5)	1.1 (0.7-1.5)	1.1 (0.7-1.5)	1.0 (0.7-1.5)	1.1 (0.7-1.6)	1.1 (0.7-1.6)	1.1 (0.8-1.7)
Fast	<100	8.2 (6.0-11.4)*	4.0 (2.5-5.1)*	3.6 (2.5-5.1)*	3.6 (2.5-5.1)*	3.3 (2.3-4.8)*	3.3 (2.2-4.8)*	2.8 (1.9-4.1)*	2.6 (1.7-3.9)*	2.4 (1.6-3.6)*	2.3 (1.5-3.6)*
	100-200	4.5 (3.3-6.2)*	2.5 (1.8-3.5)*	2.5 (1.8-3.5)*	2.5 (1.8-3.5)*	2.3 (1.6-3.3)*	2.2 (1.5-3.2)*	1.9 (1.3-2.9)*	1.9 (1.3-2.8)*	1.8 (1.2-2.7)*	1.9 (1.2-2.8)*
	200-300	2.2 (1.6-3.2)*	1.5 (1.0-2.2)*	1.5 (1.0-2.2)*	1.5 (1.0-2.1)*	1.4 (1.0-2.1)	1.6 (1.1-2.3)*	1.5 (1.0-2.3)*	1.5 (1.0-2.3)*	1.4 (0.9-2.2)	1.4 (0.9-2.2)
	300-400	1.6 (1.1-2.3)*	1.2 (0.8-1.7)	1.2 (0.8-1.7)	1.2 (0.8-1.8)	1.2 (0.8-1.9)	1.3 (0.9-2.0)	1.3 (0.9-2.0)	1.4 (0.9-2.1)	1.3 (0.9-2.0)	1.3 (0.8-2.0)
	400-500	1.3 (0.9-2.0)	1.1 (0.7-1.7)	1.1 (0.7-1.7)	1.1 (0.7-1.7)	1.1 (0.7-1.7)	1.1 (0.7-1.8)	1.1 (0.7-1.8)	1.1 (0.7-1.8)	1.1 (0.7-1.8)	1.1 (0.7-1.9)
Diesel	<100	16.3 (12.2-21.9)*	7.1 (5.1-9.8)*	7.1 (5.1-9.8)*	7.1 (5.1-9.9)*	7.0 (5.0-9.7)*	6.7 (4.8-9.5)*	6.1 (4.3-8.7)*	5.9 (4.2-8.5)*	5.4 (3.7-7.8)*	5.3 (3.6-7.6)*
	100-200	6.3 (4.7-8.4)*	3.5 (2.6-4.9)*	3.5 (2.6-4.8)*	3.5 (2.6-4.8)*	3.4 (2.4-4.6)*	3.4 (2.4-4.7)*	3.0 (2.2-4.3)*	3.1 (2.2-4.3)*	2.9 (2.1-4.2)*	2.9 (2.0-4.1)*
	200-300	2.9 (2.1-3.9)*	2.0 (1.4-2.7)*	2.0 (1.4-2.7)*	2.0 (1.4-2.7)*	1.9 (1.4-2.7)*	2.0 (1.4-2.8)*	1.9 (1.4-2.8)*	2.0 (1.4-2.8)*	1.8 (1.3-2.6)*	1.8 (1.2-2.6)*
	300-400	1.9 (1.4-2.6)*	1.4 (1.0-2.0)	1.4 (1.0-2.0)	1.4 (1.0-2.0)	1.5 (1.0-2.1)	1.5 (1.0-2.1)*	1.5 (1.0-2.2)*	1.6 (1.1-2.3)*	1.5 (1.0-2.2)*	1.5 (1.0-2.2)
	400-500	1.4 (0.9-2.0)	1.1 (0.8-1.7)	1.1 (0.8-1.7)	1.1 (0.7-1.7)	1.1 (0.7-1.6)	1.1 (0.7-1.7)	1.1 (0.7-1.6)	1.1 (0.7-1.7)	1.1 (0.7-1.7)	1.1 (0.7-1.7)
Maintenance	<100	15.2 (11.1-20.7)*	7.7 (5.5-10.8)*	7.7 (5.5-10.8)*	7.7 (5.5-10.8)*	7.6 (5.3-10.7)*	7.2 (5.1-10-4)*	6.7 (4.7-9.7)*	6.4 (4.4-9.3)*	6.0 (4.1-8.8)*	5.8 (4.0-8.5)*
	100-200	5.9 (4.3-8.0)*	3.7 (2.7-5.2)*	3.7 (2.7-5.2)*	3.7 (2.6-5.2)*	3.5 (2.5-5.0)*	3.5 (2.5-5.0)*	3.3 (2.3-4.7)*	3.2 (2.3-4.6)*	3.2 (2.2-4.6)*	3.2 (2.2-4.6)*
	200-300	3.1 (2.3-4.3)*	2.3 (1.6-3.2)*	2.3 (1.6-3.2)*	2.3 (1.6-3.2)*	2.2 (1.6-3.2)*	2.3 (1.6-3.3)*	2.2 (1.6-3.2)*	2.3 (1.6-3.3)*	2.2 (1.5-3.2)*	2.1 (1.5-3.1)*
	300-400	2.1 (1.5-2.9)*	1.7 (1.1-2.4)*	1.6 (1.1-2.4)*	1.7 (1.1-2.4)*	1.7 (1.2-2.5)*	1.7 (1.2-2.6)*	1.7 (1.2-2.6)*	1.8 (1.2-2.7)*	1.7 (1.1-2.5)*	1.6 (1.1-2.5)*
	400-500	1.6 (1.1-2.4)	1.5 (1.0-2.2)	1.5 (1.0-2.2)	1.5 (1.0-2.2)	1.5 (1.0-2.2)	1.4 (1.0-2.2)	1.4 (1.0-2.2)	1.5 (1.0.2.3)	1.5 (1.0-2.3)	1.4 (1.0-2.3)

*statistically significant
Model 1: crude analysis
Model 2: adjusting for noise annoyance
Model 3: adjusting for noise annoyance, sex
Model 4: adjusting for noise annoyance, sex, age
Model 5: adjusting for noise annoyance, sex, age, worry about health
Model 6: adjusting for noise annoyance, sex, age, worry about health, worry about safety
Model 7: adjusting for noise annoyance, sex, age, worry about health, worry about safety, worry about home
Model 8: adjusting for noise annoyance, sex, age, worry about health, worry about safety, worry about home, vibration expectation
Model 9: adjusting for noise annoyance, sex, age, worry about health, worry about safety, worry about home, vibration expectation, household income
Model 10: adjusting for noise annoyance, sex, age, worry about health, worry about safety, worry about home, vibration expectation, household income, years resident at property

Table S3. Odds of being highly annoyed by vibrations stratified by distance and train type with moderating factors (full version of Table 4).

Type of train	Distance	Model 1 OR (95%CI)	Model 2 OR (95%CI)	Model 3 OR (95%CI)	Model 4 OR (95%CI)	Model 5 OR (95%CI)	Model 6 OR (95%CI)	Model 7 OR (95%CI)	Model 8 OR (95%CI)	Model 9 OR (95%CI)	Model 10 OR (95%CI)
Passenger	<100	43.6 (10.5-180.1)*	7.0 (1.7-29.4)*	7.0 (1.7-29.4)*	7.1 (1.7-29.5)*	6.7 (1.6-28.3)*	4.5 (1.0-19.1)*	3.4 (0.8-14.8)	3.1 (0.7-13.5)	2.6 (0.6-11.5)	2.4 (0.6-10.7)
	100-200	33.0 (8.0-135.7)*	8.8 (2.1-36.5)*	8.8 (2.1-36.5)*	8.8 (2.1-36.6)*	8.5 (2.0-36.2)*	6.1 (1.4-25.6)*	5.1 (1.2-21.6)*	4.7 (1.1-20.1)*	4.2 (1.0-17.8)	4.2 (1.0-17.9)
	200-300	10.6 (2.5-45.4)*	4.1 (0.9-17.9)	4.1 (0.9-18.0)	4.1 (0.9-18.0)	3.7 (0.8-16.6)	2.9 (0.6-13.0)	2.6 (0.6-11.7)	2.6 (0.6-11.6)	2.3 (0.5-10.5)	2.3 (0.5-10.5)
	300-400	5.0 (1.1-23.5)*	2.3 (0.5-11.0)	2.3 (0.5-11.0)	2.3 (0.5-11.0)	2.5 (0.5-12.3)	1.6 (0.3-8.1)	1.5 (0.3-7.9)	1.5 (0.3-7.8)	1.3 (0.2-6.8)	1.3 (0.2-6.8)
	400-500	4.0 (0.8-20.8)	1.9 (0.3-10.4)	1.9 (0.3-10.4)	1.9 (0.3-10.4)	2.3 (0.4-13.1)	1.8 (0.3-10.1)	1.6 (0.3-9.1)	1.6 (0.3-9.1)	1.6 (0.3-9.2)	1.6 (0.3-9.2)
Freight	<100	48.7 (27.6-86.1)*	10.4 (5.8-18.8)*	10.4 (5.8-18.8)*	10.4 (5.8-18.8)*	11.9 (6.2-22.9)*	9.5 (5.0-18.0)*	7.9 (4.1-15.0)*	7.1 (3.7-13.7)*	7.9 (3.9-16.3)*	7.7 (3.8-15.8)*
	100-200	30.0 (12.4-38.7)*	7.5 (4.2-13.5)*	7.5 (4.1-13.4)*	7.5 (4.1-13-4)*	8.2 (4.3-16.7)*	6.8 (3.6-12.8)*	5.5 (2.9-10.4)*	5.1 (2.7-9.8)*	5.9 (2.9-12.1)*	5.9 (2.9-12.1)*
	200-300	5.5 (3.0-10.1)*	2.3 (1.2-4.4)*	2.3 (1.2-4.3)*	2.3 (1.2-4.3)*	2.5 (1.3-5.0)*	2.2 (1.1-4.3)*	2.0 (1.0-4.0)*	2.0 (1.0-4.0)*	2.2 (1.0-4.8)*	2.2 (1.0-4.7)*
	300-400	2.6 (1.3-5.1)*	1.3 (0.6-2.5)	1.3 (0.6-2.5)	1.3 (0.6-2.5)	1.4 (0.7-3.0)	1.2 (0.6-2.6)	1.2 (0.6-2.5)	1.2 (0.6-2.6)	1.4 (0.6-3.2)	1.4 (0.6-3.2)
	400-500	2.8 (1.4-5.5)*	1.8 (0.9-3.6)	1.7 (0.9-3.6)	1.7 (0.9-3.6)	2.2 (1.0-4.7)	1.8 (0.9-3.9)	1.7 (0.8-3.7)	1.7 (0.8-3.8)	2.0 (0.9-4.7)	2.0 (0.9-4.7)
Fast	<100	36.1 (11.3-115.9)*	6.0 (1.8-19.5)*	6.0 (1.8-19.5)*	6.0 (1.9-19.6)*	5.9 (1.8-19.6)*	3.9 (1.2-13.0)*	3.0 (0.9-10.0)	2.6 (0.8-8.8)	2.3 (0.7-7.9)	2.1 (0.6-7.3)
	100-200	24.5 (7.7-78.4)*	6.6 (2.0-21.4)*	6.6 (2.0-21.4)*	6.6 (2.0-21.5)*	6.2 (1.9-20.6)*	4.5 (1.4-14.8)*	3.7 (1.1-12.3)*	3.4 (1.0-11.2)*	3.2 (1.0-10.7)	3.1 (0.9-10.3)
	200-300	8.2 (2.5-27.6)*	3.3 (1.0-11.1)	3.3 (1.0-11.1)	3.3 (1.0-11.2)	3.2 (0.9-11.2)	2.4 (0.7-8.4)	2.2 (0.6-7.6)	2.1 (0.6-7.5)	2.0 (0.6-7.2)	2.0 (0.6-7.1)
	300-400	4.2 (1.2-15.3)*	1.9 (0.5-7.2)	1.9 (0.5-7.2)	2.0 (0.5-7.3)	2.2 (0.6-8.2)	1.5 (0.4-5.8)	1.4 (0.4-5.6)	1.4 (0.4-5.6)	1.1 (0.3-4.6)	1.1 (0.3-4.6)
	400-500	3.3 (0.8-13.0)	1.6 (0.4-6.7)	1.6 (0.4-6.7)	1.6 (0.4-6.7)	2.0 (0.5-8.5)	1.6 (0.4-6.7)	1.4 (0.3-6.1)	1.4 (0.3-6.1)	1.4 (0.3-6.3)	1.4 (0.3-6.2)
Diesel	<100	212.3 (52.5-859.1)*	43.5 (10.6-177.5)*	43.7 (10.7-178.4)*	43.8 (10.7-178.9)*	42.9 (10.4-177.1)*	33.3 (8.1-136.8)*	28.0 (6.8-115.3)*	25.2 (6.1-104.2)*	23.6 (5.7-97.9)*	22.7 (5.5-94.2)*
	100-200	84.7 (20.9-343.1)*	26.9 (6.6-109.8)*	26.8 (6.6-109.7)*	26.9 (6.6-109.8)*	25.7 (6.2-106.2)*	20.8 (5.1-85.6)*	17.6 (4.3-72.5)*	16.3 (4.0-67.4)*	15.2 (3.7-62.8)*	15.0 (3.6-62.1)*
	200-300	21.9 (5.3-90.8)*	9.6 (2.3-40.1)*	9.3 (2.2-39.2)*	9.3 (2.2-39.2)*	9.4 (2.2-40.1)*	8.3 (2.0-35.0)*	7.4 (1.8-31.4)*	7.4 (1.8-31.6)*	7.1 (1.7-30.2)*	7.1 (1.7-30.1)*
	300-400	10.8 (2.5-46.8)*	5.4 (1.2-23.7)*	5.4 (1.2-23.7)*	5.4 (1.2-23.8)*	5.9 (1.3-26.2)*	4.4 (1.0-19.5)	4.4 (1.0-19.5)	4.4 (1.0-19.7)	4.6 (1.0-20.8)*	4.6 (1.0-20.8)*
	400-500	9.0 (2.0-40.7)*	5.1 (1.1-23.7)*	5.1 (1.1-23.5)*	5.1 (1.1-23.5)*	5.6 (1.2-26.2)*	4.5 (1.0-21.0)	4.0 (0.9-19.2)	4.1 (0.9-19.3)	4.0 (0.8-19.3)	4.0 (0.8-19.0)
Maintenance	<100	57.2 (23.3-140.7)*	13.6 (5.4-33.8)*	13.7 (5.5-34.1)*	13.7 (5.5-34.2)*	13.0 (5.2-32.5)*	10.8 (4.3-27.3)*	9.8 (3.9-24.8)*	8.7 (3.4-22.0)*	8.2 (3.2-21.0)*	8.0 (3.1-20.3)*
	100-200	25.6 (10.4-63.2)*	9.0 (3.6-22.4)*	8.9 (3.6-22.3)*	8.9 (3.6-22.3)	8.2 (3.3-20.6)*	7.1 (2.8-18.0)*	6.5 (2.6-16.5)*	6.0 (2.4-15.2)*	5.8 (2.3-14.8)*	5.8 (2.3-14.7)*
	200-300	11.2 (4.4-28.1)*	5.3 (2.1-13.6)*	5.1 (2.0-13.1)*	5.1 (2.0-13.1)*	4.9 (1.9-12.7)*	4.7 (1.8-12.1)*	4.6 (1.8-11.8)*	4.5 (1.7-11.5)*	4.4 (1.7-11.4)*	4.4 (1.7-11.3)*
	300-400	5.3 (2.0-14.2)*	2.8 (1.0-7.5)*	2.8 (1.0-7.5)*	2.8 (1.0-7.5)*	2.9 (1.1-7.8)*	2.5 (0.9-6.9)	2.5 (0.9-6.9)	2.5 (0.9-6.9)	2.5 (0.9-7.0)	2.5 (0.9-6.9)
	400-500	3.9 (1.4-11.3)*	2.6 (0.9-7.6)*	2.6 (0.9-7.4)	2.6 (0.9-7.5)	2.5 (0.9-7.4)	2.1 (0.7-6.3)	2.1 (0.7-6.1)	2.0 (0.7-6.1)	2.1 (0.7-6.1)	2.0 (0.7-6.1)

*statistically significant
Model 1: crude analysis
Model 2: adjusting for noise annoyance
Model 3: adjusting for noise annoyance, sex
Model 4: adjusting for noise annoyance, sex, age
Model 5: adjusting for noise annoyance, sex, age, worry about health
Model 6: adjusting for noise annoyance, sex, age, worry about health, worry about safety
Model 7: adjusting for noise annoyance, sex, age, worry about health, worry about safety, worry about home
Model 8: adjusting for noise annoyance, sex, age, worry about health, worry about safety, worry about home, vibration expectation
Model 9: adjusting for noise annoyance, sex, age, worry about health, worry about safety, worry about home, vibration expectation, household income
Model 10: adjusting for noise annoyance, sex, age, worry about health, worry about safety, worry about home, vibration expectation, household income, years resident at property
